# 4-(2-Fluoro­pyridin-5-yl)phenol

**DOI:** 10.1107/S1600536812026499

**Published:** 2012-06-16

**Authors:** Fazal Elahi, Muhammad Adeel, M. Nawaz Tahir

**Affiliations:** a Department of Chemistry, Gomal University, Dera Ismail Khan, K.P.K., Pakistan; b University of Sargodha, Department of Physics, Sargodha, Pakistan

## Abstract

In the title compound, C_11_H_8_FNO, the aromatic rings are oriented at a dihedral angle of 31.93 (6)°. In the crystal, mol­ecules are linked by O—H⋯N hydrogen bonds, forming *C*(9) chains propagating along the *c*-axis direction. There are aromatic π–π stacking inter­actions between the pyridine rings [centroid–centroid separation = 3.7238 (16) Å].

## Related literature

For related structures, see: Adeel *et al.* (2012 ▶); Elahi *et al.* (2012 ▶).
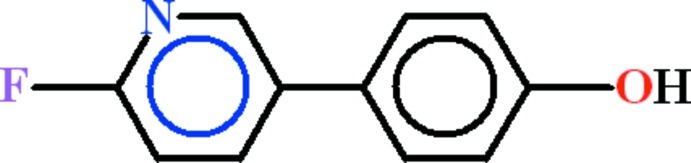


## Experimental

### 

#### Crystal data

C_11_H_8_FNO
*M* *_r_* = 189.18
Orthorhombic, 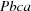

*a* = 12.275 (3) Å
*b* = 7.4343 (11) Å
*c* = 19.328 (3) Å
*V* = 1763.8 (6) Å^3^
*Z* = 8
Mo *K*α radiation
μ = 0.11 mm^−1^
*T* = 296 K
0.28 × 0.22 × 0.18 mm

#### Data collection

Bruker Kappa APEXII CCD diffractometer
Absorption correction: multi-scan (*SADABS*; Bruker, 2005 ▶) *T*_min_ = 0.975, *T*_max_ = 0.985
7508 measured reflections
1732 independent reflections
896 reflections with *I* > 2σ(*I*)
*R*_int_ = 0.064

#### Refinement

*R*[*F* ^2^ > 2σ(*F* ^2^)] = 0.051
*wR*(*F* ^2^) = 0.123
*S* = 1.00
1732 reflections
128 parameters
H-atom parameters constrained
Δρ_max_ = 0.18 e Å^−3^
Δρ_min_ = −0.18 e Å^−3^

### 

Data collection: *APEX2* (Bruker, 2007 ▶); cell refinement: *SAINT* (Bruker, 2007 ▶); data reduction: *SAINT*; program(s) used to solve structure: *SHELXS97* (Sheldrick, 2008 ▶); program(s) used to refine structure: *SHELXL97* (Sheldrick, 2008 ▶); molecular graphics: *ORTEP-3 for Windows* (Farrugia, 1997 ▶) and *PLATON* (Spek, 2009 ▶); software used to prepare material for publication: *WinGX* (Farrugia, 1999 ▶) and *PLATON*.

## Supplementary Material

Crystal structure: contains datablock(s) global, I. DOI: 10.1107/S1600536812026499/hb6846sup1.cif

Structure factors: contains datablock(s) I. DOI: 10.1107/S1600536812026499/hb6846Isup2.hkl

Supplementary material file. DOI: 10.1107/S1600536812026499/hb6846Isup3.cml

Additional supplementary materials:  crystallographic information; 3D view; checkCIF report

## Figures and Tables

**Table 1:** Hydrogen-bond geometry (Å, °)

*D*—H⋯*A*	*D*—H	H⋯*A*	*D*⋯*A*	*D*—H⋯*A*
O1—H1⋯N1^i^	0.82	2.08	2.891 (3)	168

Symmetry code: (i) 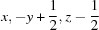.

**Fig. 1.**
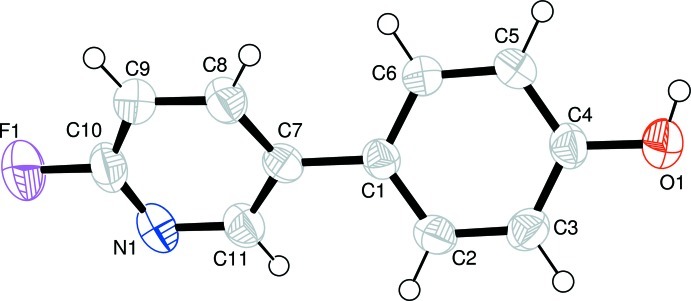
View of the title compound with displacement ellipsoids drawn at the 50% probability level.

**Fig. 2.**
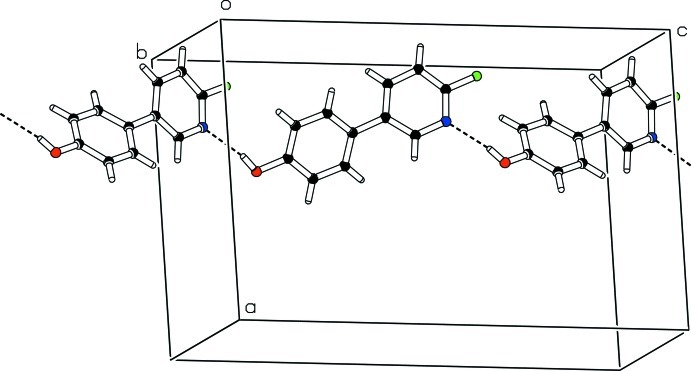
The partial packing, which shows that molecules form C(9) chains extending along [001].

## supplementary crystallographic information

### Comment

We have reported the crystal structure of 5-(4-fluorophenyl)-2-fluoropyridine (Elahi *et al.*, 2012) and 5-(4-chlorophenyl)-2-fluoropyridine (Adeel *et al.*, 2012) which are related to (I).

In (I) the 4-hydroxybenzene A (C1–C6/O1) and the 2-fluoropyridine B (C7—C11/N1/F1) are planar with r.m.s. deviations of 0.0222 Å and 0.0154 Å. The dihedral angle between A/B is 31.93 (6)°. There molecules are stabilized in the form of one-dimensional C(9) chains along the *c*-axis due to H-bondings of O—H···N type between hydroxy and pyridine groups (Table 1, Fig. 2). There exist π–π interaction between *Cg*1···*Cg*1^i^ [i = 1/2 - *x*, -1/2 + *y*, *z*] and *Cg*1···*Cg*1^ii^ [ii = 1/2 - *x*, 1/2 + *y*, *z*] at a distance of 3.7238 (16) Å, where *Cg*1 is the centroid of pyridine ring.

### Experimental

To a 6 ml solution of 5-bromo-2-fluoropyridine (0.2 g, 1.136 mmol), 4-hydroxyphenylboronic acid (0.190 g, 1.36 mmol) in dioxane and K_3_PO_4_ (0.361 g, 1.5 mmol, in 1 ml H_2_O) was added Pd(PPh_3_)_4_ (1.5 mole %) at 373 K under N_2_ atmosphere. The reaction mixture was refluxed for 8 h. Then 20 ml of distilled water was added. The aqueous layer was extracted three times with EtOAc(3×15 ml). The organic layer was evaporated in *vacuo* and title compound was obtained as light brown solid. Yield: 0.191 g, 89%. *M*.p. 350–352 K. Crystallization from a saturated solution of CHCl_3_ /CH_3_OH gave light brown plates.

### Refinement

The H-atoms were positioned geometrically (C–H = 0.93, O—H = 0.82 Å) and refined as riding with *U*_iso_(H) = *xU*_eq_(C, O), where *x* = 1.5 for hydroxy and *x* = 1.2 for other H-atoms.

### Crystal data

**Table:**

C_11_H_8_FNO	*F*(000) = 784
*M**_r_* = 189.18	*D*_x_ = 1.425 Mg m^−^^3^
Orthorhombic, *P**b**c**a*	Mo *K*α radiation, λ = 0.71073 Å
Hall symbol: -P 2ac 2ab	Cell parameters from 869 reflections
*a* = 12.275 (3) Å	θ = 2.1–26.0°
*b* = 7.4343 (11) Å	µ = 0.11 mm^−^^1^
*c* = 19.328 (3) Å	*T* = 296 K
*V* = 1763.8 (6) Å^3^	Plate, light brown
*Z* = 8	0.28 × 0.22 × 0.18 mm

### Data collection

**Table:**

Bruker Kappa APEXII CCD diffractometer	1732 independent reflections
Radiation source: fine-focus sealed tube	896 reflections with *I* > 2σ(*I*)
Graphite monochromator	*R*_int_ = 0.064
Detector resolution: 8.00 pixels mm^-1^	θ_max_ = 26.0°, θ_min_ = 2.1°
ω scans	*h* = −15→6
Absorption correction: multi-scan (*SADABS*; Bruker, 2005)	*k* = −9→9
*T*_min_ = 0.975, *T*_max_ = 0.985	*l* = −17→23
7508 measured reflections	

### Refinement

**Table:**

Refinement on *F*^2^	Primary atom site location: structure-invariant direct methods
Least-squares matrix: full	Secondary atom site location: difference Fourier map
*R*[*F*^2^ > 2σ(*F*^2^)] = 0.051	Hydrogen site location: inferred from neighbouring sites
*wR*(*F*^2^) = 0.123	H-atom parameters constrained
*S* = 1.00	*w* = 1/[σ^2^(*F*_o_^2^) + (0.0483*P*)^2^] where *P* = (*F*_o_^2^ + 2*F*_c_^2^)/3
1732 reflections	(Δ/σ)_max_ < 0.001
128 parameters	Δρ_max_ = 0.18 e Å^−^^3^
0 restraints	Δρ_min_ = −0.18 e Å^−^^3^

### Special details

**Table:**

Geometry. Bond distances, angles *etc*. have been calculated using the rounded fractional coordinates. All su's are estimated from the variances of the (full) variance-covariance matrix. The cell e.s.d.'s are taken into account in the estimation of distances, angles and torsion angles
Refinement. Refinement of *F*^2^ against ALL reflections. The weighted *R*-factor *wR* and goodness of fit *S* are based on *F*^2^, conventional *R*-factors *R* are based on *F*, with *F* set to zero for negative *F*^2^. The threshold expression of *F*^2^ > σ(*F*^2^) is used only for calculating *R*-factors(gt) *etc*. and is not relevant to the choice of reflections for refinement. *R*-factors based on *F*^2^ are statistically about twice as large as those based on *F*, and *R*- factors based on ALL data will be even larger.

### Fractional atomic coordinates and isotropic or equivalent isotropic displacement parameters (Å^2^)

**Table:**

	*x*	*y*	*z*	*U*_iso_*/*U*_eq_	
F1	0.16138 (14)	0.0574 (2)	0.57218 (6)	0.0807 (7)	
O1	0.44529 (15)	0.4234 (3)	0.12632 (8)	0.0611 (8)	
N1	0.3071 (2)	0.1137 (3)	0.50603 (9)	0.0532 (9)	
C1	0.3319 (2)	0.2565 (3)	0.31870 (11)	0.0361 (9)	
C2	0.4308 (2)	0.3444 (3)	0.31099 (12)	0.0414 (9)	
C3	0.4687 (2)	0.3971 (3)	0.24670 (12)	0.0439 (10)	
C4	0.4071 (2)	0.3622 (3)	0.18829 (12)	0.0416 (9)	
C5	0.3108 (2)	0.2682 (3)	0.19463 (11)	0.0449 (10)	
C6	0.2736 (2)	0.2170 (3)	0.25895 (10)	0.0419 (9)	
C7	0.2856 (2)	0.2081 (3)	0.38699 (11)	0.0367 (9)	
C8	0.1738 (2)	0.2085 (3)	0.39711 (12)	0.0462 (10)	
C9	0.1293 (2)	0.1575 (3)	0.45939 (12)	0.0520 (11)	
C10	0.2008 (3)	0.1116 (3)	0.51007 (12)	0.0524 (10)	
C11	0.3492 (2)	0.1610 (3)	0.44353 (11)	0.0470 (10)	
H1	0.39877	0.40831	0.09643	0.0916*	
H2	0.47273	0.36848	0.34999	0.0497*	
H3	0.53530	0.45572	0.24276	0.0527*	
H5	0.27072	0.23925	0.15533	0.0540*	
H6	0.20815	0.15470	0.26247	0.0503*	
H8	0.12817	0.24378	0.36118	0.0555*	
H9	0.05436	0.15458	0.46643	0.0624*	
H11	0.42453	0.16170	0.43841	0.0565*	

### Atomic displacement parameters (Å^2^)

**Table:**

	*U*^11^	*U*^22^	*U*^33^	*U*^12^	*U*^13^	*U*^23^
F1	0.0964 (16)	0.1031 (13)	0.0427 (9)	−0.0147 (11)	0.0132 (9)	0.0107 (8)
O1	0.0498 (15)	0.0910 (14)	0.0424 (10)	−0.0135 (11)	0.0072 (9)	0.0057 (10)
N1	0.066 (2)	0.0596 (15)	0.0339 (13)	0.0057 (14)	0.0004 (12)	−0.0012 (10)
C1	0.0395 (18)	0.0352 (13)	0.0335 (14)	0.0013 (12)	0.0020 (12)	−0.0030 (10)
C2	0.0406 (18)	0.0456 (14)	0.0380 (14)	0.0002 (13)	−0.0052 (12)	−0.0044 (10)
C3	0.0363 (18)	0.0471 (16)	0.0484 (16)	−0.0038 (13)	0.0056 (13)	−0.0035 (11)
C4	0.0421 (19)	0.0493 (15)	0.0335 (15)	0.0028 (14)	0.0063 (13)	−0.0012 (11)
C5	0.045 (2)	0.0555 (16)	0.0341 (15)	−0.0048 (14)	−0.0050 (12)	−0.0024 (11)
C6	0.0418 (18)	0.0457 (15)	0.0382 (15)	−0.0071 (13)	0.0006 (13)	−0.0018 (11)
C7	0.0412 (19)	0.0336 (13)	0.0354 (14)	0.0023 (12)	−0.0028 (13)	−0.0028 (10)
C8	0.049 (2)	0.0521 (16)	0.0376 (16)	0.0036 (14)	−0.0005 (14)	0.0011 (11)
C9	0.048 (2)	0.0627 (18)	0.0453 (17)	−0.0060 (15)	0.0049 (15)	−0.0046 (12)
C10	0.069 (2)	0.0544 (17)	0.0339 (17)	−0.0055 (17)	0.0102 (17)	−0.0002 (12)
C11	0.050 (2)	0.0504 (16)	0.0405 (16)	0.0029 (14)	0.0015 (14)	−0.0054 (11)

### Geometric parameters (Å, º)

**Table:**

F1—C10	1.356 (3)	C7—C8	1.386 (3)
O1—C4	1.364 (3)	C7—C11	1.388 (3)
O1—H1	0.8200	C8—C9	1.375 (3)
N1—C11	1.360 (3)	C9—C10	1.359 (4)
N1—C10	1.307 (4)	C2—H2	0.9300
C1—C2	1.387 (3)	C3—H3	0.9300
C1—C7	1.481 (3)	C5—H5	0.9300
C1—C6	1.390 (3)	C6—H6	0.9300
C2—C3	1.384 (3)	C8—H8	0.9300
C3—C4	1.383 (3)	C9—H9	0.9300
C4—C5	1.379 (3)	C11—H11	0.9300
C5—C6	1.378 (3)		
			
C4—O1—H1	109.00	N1—C10—C9	126.8 (2)
C10—N1—C11	115.8 (2)	F1—C10—C9	118.9 (3)
C2—C1—C6	117.5 (2)	N1—C11—C7	123.4 (2)
C6—C1—C7	119.4 (2)	C1—C2—H2	119.00
C2—C1—C7	123.1 (2)	C3—C2—H2	119.00
C1—C2—C3	121.6 (2)	C2—C3—H3	120.00
C2—C3—C4	119.7 (2)	C4—C3—H3	120.00
O1—C4—C5	122.8 (2)	C4—C5—H5	120.00
C3—C4—C5	119.4 (2)	C6—C5—H5	120.00
O1—C4—C3	117.8 (2)	C1—C6—H6	119.00
C4—C5—C6	120.3 (2)	C5—C6—H6	119.00
C1—C6—C5	121.4 (2)	C7—C8—H8	119.00
C1—C7—C8	120.3 (2)	C9—C8—H8	119.00
C1—C7—C11	123.2 (2)	C8—C9—H9	122.00
C8—C7—C11	116.5 (2)	C10—C9—H9	122.00
C7—C8—C9	121.1 (2)	N1—C11—H11	118.00
C8—C9—C10	116.3 (2)	C7—C11—H11	118.00
F1—C10—N1	114.4 (2)		
			
C11—N1—C10—F1	177.63 (19)	C2—C3—C4—O1	177.2 (2)
C11—N1—C10—C9	−2.5 (4)	C2—C3—C4—C5	−2.8 (3)
C10—N1—C11—C7	1.2 (3)	O1—C4—C5—C6	−176.9 (2)
C6—C1—C2—C3	2.3 (3)	C3—C4—C5—C6	3.1 (3)
C7—C1—C2—C3	−176.1 (2)	C4—C5—C6—C1	−0.7 (4)
C2—C1—C6—C5	−2.0 (3)	C1—C7—C8—C9	177.6 (2)
C7—C1—C6—C5	176.5 (2)	C11—C7—C8—C9	−2.6 (3)
C2—C1—C7—C8	146.8 (2)	C1—C7—C11—N1	−178.9 (2)
C2—C1—C7—C11	−33.1 (3)	C8—C7—C11—N1	1.2 (3)
C6—C1—C7—C8	−31.6 (3)	C7—C8—C9—C10	1.5 (3)
C6—C1—C7—C11	148.5 (2)	C8—C9—C10—F1	−178.9 (2)
C1—C2—C3—C4	0.1 (3)	C8—C9—C10—N1	1.2 (4)

### Hydrogen-bond geometry (Å, º)

**Table:**

*D*—H···*A*	*D*—H	H···*A*	*D*···*A*	*D*—H···*A*
O1—H1···N1^i^	0.82	2.08	2.891 (3)	168

Symmetry code: (i) *x*, −*y*+1/2, *z*−1/2.
